# Supplementary material for: Smaller infarct size with ticagrelor vs. clopidogrel in STEMI patients: Insights from cardiac magnetic resonance
Source: PLoS One. 2025 Oct 27;20(10):e0328114. doi: 10.1371/journal.pone.0328114 (PMC12558520; doi:10.1371/journal.pone.0328114)
Supplement: S1 File — Laboratory parameters did not differ between antiplatelet groups at 30 days after STEMI. (DOCX) [file pone.0328114.s001.docx]

Table S1. Laboratory parameters after 30 days of STEMI

|  | Ticagrelor | Clopidogrel | P value |
| --- | --- | --- | --- |
| Total cholesterol | 120 (105-144) | 127 (106-153) | 0.48 |
| LDL-cholesterol | 57 (46-78) | 65 (47-86) | 0.21 |
| HDL-cholesterol | 39 (34-46) | 37 (31-43) | 0.053 |
| Triglycerides | 131 (98-172) | 119 (97-161) | 0.22 |
| Non HDL-cholesterol | 83 (67-105) | 87 (69-113) | 0.29 |
| Creatinine | 0.94 (0.81-1.24) | 0.93 (0.83-1.09) | 0.73 |
| Glucose | 99 (91-116) | 98 (90-111) | 0.31 |

Values are mg/dL. Comparisons were made by the Mann-Whitney U test.
