# Supplementary material for: Smaller infarct size with ticagrelor vs. clopidogrel in STEMI patients: Insights from cardiac magnetic resonance
Source: PLoS One. 2025 Oct 27;20(10):e0328114. doi: 10.1371/journal.pone.0328114 (PMC12558520; doi:10.1371/journal.pone.0328114)
Supplement: S2 File — This project aimed to compare the effects of lipid-lowering and antiplatelet therapies (2x2 factorial design) on the infarct size and ventricular remodeling (CMR). Researchers from Universidade Federal de São Paulo, Universidade Estadual de Campinas, Universidade Santo Amaro, Universidade São Paulo, Instituto Dante Pazzanese de Cardiologia, Hospital Israelita Albert Einstein, Royal Imperial College (UK), participated in the study. (DOCX) [file pone.0328114.s002.docx]

**The B And T Types of Lymphocytes Evaluation in Acute Myocardial Infarction (BATTLE-AMI) trial investigators**

Francisco A H Fonseca (Universidade Federal de São Paulo) – Lead author

Maria Cristina O Izar (Universidade Federal de São Paulo)

Gilberto Szarf (Universidade Federal de são Paulo)

Ibraim F M Pinto (Instituto Dante Pazzanese de Cardiologia)

Carolina N França (Universidade Santo Amaro)

Amanda S F Bacchin (Universidade Santo Amaro)

Adriano M Caixeta (Universidade Federal de São Paulo)

Antonio M Figueiredo Neto (Universidade de São Paulo)

Henrique T Bianco (Universidade Federal de São Paulo)

Henrique A Fonseca (Hospital Israelita Albert Einstein)

Ana C Aguirre (Universidade Federal de São Paulo)

Flavio T Moreira (Universidade Federal de São Paulo)

Otavio Berwanger (Royal Imperial College – London)

Aline S Lopes (Universidade Federal de São Paulo)

Aline Klassen (Universidade Federal de São Paulo)

Michelle Birtche (Universidade Federal de São Paulo)

Igor Batista (Universidade Federal de São Paulo)

Juliana Kato (Universidade Santo Amaro)

Ieda L Maugeri (Universidade Federal de São Paulo)

Marina Tavares (Universidade de São Paulo)

Antonio C C Carvalho (Universidade Federal de São Paulo)

Mario A Saad (Universidade Estadual de Campinas)

Weverton Leite (Universidade Federal de São Paulo)

Carlos E Ferreira (Hospital Israelita Albert Einstein)

Nayara M Okazaki (Universidade Federal de São Paulo)

Maria E R Coste (Universidade Federal de São Paulo)

Iran Gonçalves Jr (Universidade Federal de São Paulo)
