# Supplementary material for: Smaller infarct size with ticagrelor vs. clopidogrel in STEMI patients: Insights from cardiac magnetic resonance
Source: PLoS One. 2025 Oct 27;20(10):e0328114. doi: 10.1371/journal.pone.0328114 (PMC12558520; doi:10.1371/journal.pone.0328114)
Supplement: Attachment — Submitted filename: Responses to reviewers.docx [file pone.0328114.s005.docx]

Responses to reviewers

Reviewer #1:

The manuscript presents a well-designed randomized clinical trial comparing the effects of ticagrelor and clopidogrel on infarct size in STEMI patients managed with a pharmaco-invasive strategy. The manuscript is technically robust. The study design adheres to rigorous clinical trial standards. The data strongly support the primary conclusion that ticagrelor is associated with a smaller infarct size compared to clopidogrel in STEMI patients, including the primary outcome of a significantly smaller percentage of left ventricular infarcted mass (p=0.012), with consistent results in both absolute (grams) and relative (%) measurements. Despite similar baseline angiographic characteristics (Syntax score, TIMI flow, culprit artery distribution) and post-PCI reperfusion success, the ticagrelor group showed more homogeneous responses (via K-means clustering) with smaller infarct sizes and better LVEF. Lower high-sensitivity troponin T (hsTNT) and high-sensitivity C-reactive protein (hsCRP) on day 1 in the ticagrelor group suggest early benefits on microcirculation, reinforcing the mechanistic link to reduced infarct size. Confounding variables (e.g., lipid profiles, other inflammatory markers) were balanced between groups, strengthening the causal inference. The statistical approach is rigorous and appropriate for the study design. The manuscript is well-structured and written in clear, standard English. Tables and figures are informative and support the narrative, though legends could be slightly expanded to clarify statistical significance markers for non-specialist readers.

Authors: we added information in the legend of figure 2.

Figure 2. Box plots illustrating differences between the ticagrelor and clopidogrel groups on the first day of myocardial infarction. A – High-sensitivity troponin (hsTNT); B – High-sensitivity C-reactive protein (hsCRP). Lower levels of hsTNT and hsCRP were observed among patients treated with ticagrelor compared to those treated with clopidogrel (Mann-Whitney test).

The study is limited to STEMI patients <75 years without prior myocardial infarction, managed via a pharmaco-invasive strategy. Caution is advised when extrapolating to primary PCI populations, which the authors appropriately note in limitations. The discussion links ticagrelor's benefits to improved microcirculation (via adenosine uptake inhibition) and reduced inflammation, supported by prior literature. Expanding on how improved LDL quality (cited in ref. 39) might contribute to long-term outcomes could strengthen the mechanistic narrative. While the sample (n=225) is sufficient to detect differences in infarct size, larger trials are warranted to validate these findings, as acknowledged in the conclusion.
This manuscript is methodologically sound, with robust data supporting its conclusions. The rigorous design, appropriate use of CMR, and thorough statistical analysis make it a valuable contribution to understanding antiplatelet therapy in STEMI. Minor refinements to mechanistic discussions and generalizability notes would further enhance its impact. We recommend consideration for publication.

Authors: The authors appreciate your comments. We added minor refinements to mechanistic discussions and generalizability notes to improve the manuscript (see page 10 lines 352-359).

In the PLATO study, the primary endpoint (cardiovascular death, non-fatal myocardial infarction, or non-fatal stroke) was significantly reduced even in the period after the first 30 days up to one year^1^. These data suggest continued benefits in addition to those directly related to thrombosis or microcirculation during the acute phase of myocardial infarction. Our results were obtained in patients undergoing pharmaco-invasive strategy, the only group of acute coronary syndrome patients not included in the PLATO study^1^.

Reviewer #2:

Interesting paper. Some issues should be addressed

Major
Do authors think that these findings hold true for patients treated with pPCI without thrombolysis?
Time of enrollment compared to number of patients appears long. Please comment
Abstract: please add numeric values for the area at risk
Recently length and kind of DAPT demonstrated to change prognosis. Please relate your findings with those from a clinical point of view (quote on PMID: 38242567)

Authors: We appreciate your comments.

Do authors think that these findings hold true for patients treated with pPCI without thrombolysis?

Authors: It is an important question (see page 10 lines 352-362).

In the PLATO study, the primary endpoint (cardiovascular death, non-fatal myocardial infarction, or non-fatal stroke) was significantly reduced even in the period after the first 30 days up to one year^1^. These data suggest continued benefits in addition to those directly related to thrombosis or microcirculation during the acute phase of myocardial infarction. Our results were obtained in patients undergoing pharmaco-invasive strategy, the only group of acute coronary syndrome patients not included in the PLATO study^1^. Later, in the TREAT trial^10^, the safety for bleeding in patients following pharmaco-invasive strategy was similar between those receiving ticagrelor or clopidogrel. However, the study did not have sufficient power to analyze major cardiovascular endpoints^10^. Therefore, for patients undergoing pharmaco-invasive strategy, despite promising data in our study, larger studies addressing cardiovascular outcomes are needed.

Time of enrollment compared to number of patients appears long. Please comment

Authors: In fact, we included patients from May 2015 to March 2020. This study is part of the BATTLE-AMI trial, a thematic study addressing the key players in the amount of infarcted mass and LVEF quantified by CMR. In the trial protocol we included only stable patients undergoing pharmacological thrombolysis in the first 6 hours of symptoms onset and referred to our university hospital in the first 24 hours for coronary angiography and invasive procedures. Based on the inclusion and exclusion criteria, we gradually reached approximately the planned number of patients only in March 2020. At this point we had the challenge of the coronavirus epidemic and we decided to end the study. (see pages 3 lines 99-126).

Abstract: please add numeric values for the area at risk
Recently length and kind of DAPT demonstrated to change prognosis. Please relate your findings with those from a clinical point of view (quote on PMID: 38242567)

Authors: unfortunately we did not measure the area at risk by CMR, however the groups were comparable for both pre- and post-PCI, and for most clinical and laboratory data. Area at risk is an important topic and we will include it in future manuscripts using a new software for this analysis.

The length and kind of DAPT is really a very important issue. We added two recent meta-analyses addressing bleeding rates and ischemic events among ACS patients with de-escalation of DAPT. (see page 10,11, lines 364-372; ref 40 and 41).

More recently, DAPT strategies and de-escalation after 1-3 months have been examined. First, a comparison of six antiplatelet strategies in ACS patients undergoing PCI was reported, showing lower bleeding rates with de-escalation of DAPT without increase in stent thrombosis^40^. Further, meta-analysis of six trials showed that compared with 12-months of DAPT, de-escalation (after 1-3 months of DAPT), with ticagrelor as a single antiplatelet agent, reduced bleeding rates and did not increase ischemic events. In fact, DAPT de-escalation was associated with decrease in cardiovascular and all-cause mortality^41^.

The authors appreciate your valuable comments.
